# Leveraging Education Science for AI-Clinician Collaboration in the Patient Care Ecosystem

**DOI:** 10.1097/AS9.0000000000000442

**Published:** 2024-05-13

**Authors:** Martin Grønnebæk Tolsgaard, Aasa Feragen, Lawrence Grierson

**Affiliations:** * From the Copenhagen Academy for Medical Education and Simulation (CAMES), Copenhagen, Denmark; † Department of Obstetrics, Copenhagen University Hospital Rigshospitalet, Copenhagen, Denmark; ‡ Department of Medicine, University of Copenhagen, Copenhagen, Denmark; § DTU Compute, Technical University of Denmark, Lyngby, Denmark; ‖ Department of Family Medicine, McMaster University, Hamilton, Ontario, Canada.

The discourse of artificial intelligence (AI) in the medical practice literature emphasizes its great promise for advancing healthcare by changing the ways that clinical tasks are performed.^1^ However, multiple studies have documented the malalignment of AI development with clinician end-user needs, thereby reducing the impact of AI on healthcare delivery.^2^

Here, it’s helpful to recognize the patient, AI, and physician as part of a dynamic healthcare ecosystem, in which all 3 must work together. Clinicians are not one-size-fits-all—they are individuals who are managing the proliferation of a dynamic skill set; continuous life-long learners who enter the clinical space with important personal education objectives. To date, AI for healthcare research does not typically take the science of human learning into account when conceptualizing, designing, and evaluating AI in the healthcare ecosystem.^3^ Yet, there are several looming education tensions that need to be addressed when it comes to introducing AI into healthcare contexts. The introduction of AI may promote the widespread degradation or loss of fundamental clinical skills. For instance, by providing a diagnosis that the physician does not need to reason first, the AI may disrupt the physician’s ability to consolidate the deep conceptual knowledge that underpins clinical decision-making. This may be particularly damaging when one considers the mounting evidence that these deep knowledge structures are essential to the way physicians innovate solutions in the face of unique healthcare challenges.^4^ If AI degrades the physician’s conceptual knowledge base, then it may also corrupt the ability to innovate solutions to new problems and advance the field.

The absence of education theory and science in the AI for healthcare literature resembles earlier periods in history when new technology for clinicians was introduced. Indeed, technological hype in healthcare often precedes the generation of an evidence base to guide the adoption of the new technology. For example, robot-assisted surgery was implemented in the context of lacking and, at times, conflicting evidence to support its benefits for patients.^5^ The integration of simulation-based learning technologies for medical education also outpaced adequate consideration for learning theory, leading to frivolous spending and the influx of many simulators that do not actually elevate clinician performance.^6^

Fortunately, AI research in the context of general education science offers more perspective on how these systems may be employed in service of learning objectives.^7^ Here, the typical AI application involves systems that determine learning challenges that are customized to the learner’s current level of domain-relevant knowledge, inspiring efforts that foster the progression toward expertise in a step-based fashion.^8^ For example, a personalized AI would take task complexity and level of expertise into consideration when generating questions, tailoring feedback, and providing explanations. This has given way to the burgeoning field of learning analytics, which is concerned with the collection and analysis of data about learners and their contexts to optimize the outcomes of training and the environments in which it occurs.^9^ Research on learning analytics may offer a particularly fruitful foundation for understanding how AI systems can be constructed to ensure that the development of health professional expertise is a central consideration within the AI-clinician collaboration.

In recognizing the potential of AI for education, we also recognize that AI for healthcare systems needs to better integrate learning theory to reach its full potential. However, the current education literature does not provide strong theory-based perspectives on how to do this.^3^ The typical personalized, step-based, AI education systems operate based on pertinent education theories such as the desirable difficulties and proximal learning frameworks, which both advocate for designs that present the learners with challenges beyond the knowledge base they have already mastered. In terms of AI-powered decision support, this translates to showing different levels of feedback and information to users at different levels of clinical skill. Importantly, evidence supporting both frameworks shows that these types of challenges increase errors and slow the rate of improvement along the way to robust, well-developed skills.^10^ Of course, systems that support learning through increased error and slower rates of improvement cannot be tolerated in the context of concurrent patient care. Herein we recognize the fundamental tension in meeting both the physician’s clinical and educational objectives at the same time. AI for healthcare systems must also be adaptive learning systems that improve physicians’ knowledge, skills, and behaviors. In the long run, developments that better integrate learning theory will ultimately have a larger impact on health outcomes than AI that focuses exclusively on bolstering episodic physician performance.

## ACKNOWLEDGMENTS

The Danish Signature Project; The Pioneer Centre for Artificial Intelligence.
